# Patient Preferences for Treatment Outcomes in Oncology with a Focus on the Older Patient—A Systematic Review

**DOI:** 10.3390/cancers14051147

**Published:** 2022-02-23

**Authors:** Petronella A. L. (Nelleke) Seghers, Anke Wiersma, Suzanne Festen, Mariken E. Stegmann, Pierre Soubeyran, Siri Rostoft, Shane O’Hanlon, Johanneke E. A. Portielje, Marije E. Hamaker

**Affiliations:** 1 Department of Geriatric Medicine, Diakonessenhuis, 3582 KE Utrecht, The Netherlands; 2 Department of Internal Medicine, Diakonessenhuis, 3582 KE Utrecht, The Netherlands; ankewiersma@outlook.com; 3 University Center for Geriatric Medicine, University Medical Hospital Groningen, University of Groningen, 9713 GZ Groningen, The Netherlands; s.festen@umcg.nl; 4 Department of General Practice and Elderly Care Medicine, University Medical Center Groningen, University of Groningen, 9713 GZ Groningen, The Netherlands; m.e.stegmann@umcg.nl; 5 Department of Oncology, Institut Bergonié, Université de Bordeaux, 33076 Bordeaux, France; p.soubeyran@bordeaux.unicancer.fr; 6 Department of Geriatric Medicine, Oslo University Hospital, 0424 Oslo, Norway; siri.rostoft@medisin.uio.no; 7 Institute of Clinical Medicine, University of Oslo, 0318 Oslo, Norway; 8 Department of Geriatric Medicine, St. Vincent’s University Hospital, D04 T6F4 Dublin, Ireland; shaneohanlon@svhg.ie; 9 School of Medicine, University College Dublin, D04 V1W8 Dublin, Ireland; 10 Department of Medical Oncology, Leiden University Medical Center-LUMC, 2333 ZA Leiden, The Netherlands; j.e.a.portielje@lumc.nl

**Keywords:** patient preferences, quality of life, geriatric oncology, trade-off, cancer

## Abstract

**Simple Summary:**

In oncology, treatment outcomes can be competing, which means that one treatment could benefit one outcome, like survival, and negatively influence another, like independence. The choice of treatment therefore depends on the patient’s preference for outcomes, which needs to be assessed explicitly. Especially in older patients, patient preferences are important. Our systematic review summarizes all studies that assessed patient preferences for various treatment outcome categories. A total of 28 studies with 4374 patients were included, of which only six studies included mostly older patients. Although quality of life was only included in half of the studies, overall quality of life (79%) was most frequently prioritized as highest or second highest, followed by overall survival (67%), progression- and disease-free survival (56%), absence of severe or persistent treatment side effects (54%), treatment response (50%), and absence of transient short-term side effects (16%). In shared decision-making, these results can be used by healthcare professionals to better tailor the information provision and treatment recommendations to the individual patient.

**Abstract:**

For physicians, it is important to know which treatment outcomes are prioritized overall by older patients with cancer, since this will help them to tailor the amount of information and treatment recommendations. Older patients might prioritize other outcomes than younger patients. Our objective is to summarize which outcomes matter most to older patients with cancer. A systematic review was conducted, in which we searched Embase and Medline on 22 December 2020. Studies were eligible if they reported some form of prioritization of outcome categories relative to each other in patients with all types of cancer and if they included at least three outcome categories. Subsequently, for each study, the highest or second-highest outcome category was identified and presented in relation to the number of studies that included that outcome category. An adapted Newcastle–Ottawa Scale was used to assess the risk of bias. In total, 4374 patients were asked for their priorities in 28 studies that were included. Only six of these studies had a population with a median age above 70. Of all the studies, 79% identified quality of life as the highest or second-highest priority, followed by overall survival (67%), progression- and disease-free survival (56%), absence of severe or persistent treatment side effects (54%), and treatment response (50%). Absence of transient short-term side effects was prioritized in 16%. The studies were heterogeneous considering age, cancer type, and treatment settings. Overall, quality of life, overall survival, progression- and disease-free survival, and severe and persistent side effects of treatment are the outcomes that receive the highest priority on a group level when patients with cancer need to make trade-offs in oncologic treatment decisions.

## 1. Introduction

Being diagnosed with cancer is a major life event and the start of a complex decision-making process on cancer treatment. Often, several treatment options are available. Most cancer treatments are intensive and burdensome, and the outcome cannot be guaranteed [1,2]. Furthermore, outcomes can be competing. For example, adjuvant chemotherapy in stage III colon cancer may decrease the likelihood of recurrence and increase (cancer-specific) survival, but toxicity may impact quality of life in the short term while serious treatment-related complications could also impact long-term functioning. Trade-offs are therefore needed.

The gold standard for complex decisions in oncology is shared decision-making, of which an important step is explicitly discussing which outcomes matter most to the patient [3,4,5,6]. This is particularly relevant in older patients, who may have a less favorable balance of benefits and risks of treatment than younger patients [1,7,8,9]. They are often excluded from clinical trials, and as a consequence, their recommendations are less evidence based [10]. Furthermore, oncological treatments have a narrow therapeutic index between the possible benefit of cancer control, including cancer symptom reduction, and the price that is still considered acceptable in terms of side effects. This increases the uncertainty in decision-making and makes it even more important to know which treatment outcomes are most frequently prioritized by older patients with cancer.

Knowledge of the most frequently mentioned patient priorities allows for a tailoring of information provision and prevents information overload caused by summing up all the treatment and outcome possibilities during the shared decision-making [3,4,5,6]. Prior research has demonstrated that adequate information provision about treatment impact and adverse events reduces the likelihood of decision regret [11] and improves patient satisfaction [12].

In patient preference elicitation many methods exist. Some methods are more general and ask patients to explicitly indicate what they would prefer, like rating scales [13] or the Outcome Prioritization Tool (OPT), which explicitly asks patients to rate each outcome relative to other outcomes without having two values on the same level [14]. This uses a trade-off principle: By prioritizing one outcome, patients are willing to accept the deterioration of other outcomes. The outcomes that are assigned priorities in the OPT conversation include extending life, maintaining independence, reducing pain, and reducing other symptoms [15]. Other methods are more specific and implicit, like discrete choice experiment (DCE), conjoint analysis (CA), and probability trade-off (Trade-off). These methods present patients with hypothetical scenarios with information on the possible benefits and side effects that are associated with various treatments and the probability of those happening. By measuring the willingness of patients to choose a treatment option while providing different scenarios of the included variables, the relative importance of that variable can be calculated [13]. Furthermore, the analytic hierarchy process (AHP) gives patients pair-wise comparisons and asks them to rate them against each other. This is also leads to a calculated relative importance of all included variables [16]. All methods have their benefits, and the best method of preference assessment depends on the question it needs to answer and the (number of) trade-offs that are at stake [13].

Two types of treatment outcomes are described in the literature [17,18]: disease-centered outcomes, which measure the objective effect of the treatment on the tumor and the adverse events, such as treatment response, toxicity, and disease-free survival, and patient-centered outcomes, which focus on the patient’s perception of health, quality of life, and functional outcomes like maintaining independence. To get a complete overview of the patient priorities in older patients with cancer, we set out to gather all available evidence from trade-off studies regarding treatment outcomes (both disease-centered outcomes and patient-centered outcomes).

## 2. Materials and Methods

We performed a systematic review to collect all available quantitative evidence comparing the relative importance patients allocate to various patient- and disease-centered outcomes after a cancer diagnosis. During the process the PRISMA guidelines were used [19]. We registered the systematic review in the OSF registry from the Center for Open Science [20].

### 2.1. Search Strategy

On 22 December 2020, we performed a search in Embase and Medline with the following terms and their synonyms: “health or treatment outcomes,” “priorities,” “trade-offs,” and “cancer.” The full electronic search strategy is shown in Appendix A. The search was limited to studies on humans written in English and published in the past 15 years. After an initial search in older patients, which resulted in few specific data, the search was expanded to all ages.

The titles and abstracts of all studies retrieved by the searches were assessed by one reviewer (N.S.) to determine which ones warranted further examination. All potentially relevant titles were subsequently screened independently as full text by two reviewers (N.S., A.W.). If no full text was found, the reviewers tried to find the final report of the study by using names of the different authors in combination with key words from the title. If none were found, the studies were excluded.

### 2.2. Eligibility Criteria

We included original publications on the comparison of outcome priorities after a diagnosis of cancer; this included both studies in actual cancer patients and studies performed on other subjects asked to state their priority in the hypothetical situation of a cancer diagnosis. Studies were only included if they addressed at least three of the possible six outcome categories that were defined, which were transient short-term side effects, severe and persistent side effects, quality of life (including functioning), treatment response, progression- and disease-free survival, and overall survival (see Appendix B).

All methods of preference elicitation were allowed, as long as the studies provided a form of prioritization of the individual outcome categories. Therefore, studies were excluded if the relative importance of outcome categories could not be elucidated due to the way the results were elicited or reported.

### 2.3. Data Extraction

Both reviewers (N.S., A.W.) independently extracted the following characteristics: title, author, year of publication, country, cancer type, curative or palliative treatment setting, me(di)an age of the sample, sample size, and method of assessing preferences. In addition, any patient- or disease-centered outcomes that were included in the trade-offs in the study were extracted, together with the ranking or score regarding the priority for each of these outcomes. Outcomes relating to process attributes such as mode of administration, frequency of administration, or out of pockets costs were not included.

### 2.4. Quality Assessment

Quality assessment was carried out by two independent reviewers using a quality assessment based on the Newcastle–Ottawa Scale ([21]; N.S. and A.W.), adjusted for this purpose based on a validated checklist for conjoint analysis (Appendix C) [22,23,24]. Disagreements were discussed in a consensus meeting and in case of continuing disagreement, a third reviewer (M.H.) was consulted.

### 2.5. Data Synthesis and Analysis

Based on the outcomes used by the included studies, two reviewers (N.S., A.W.) defined six outcome categories: quality of life (including functioning), overall survival, progression- and disease-free survival, severe and persistent side effects of treatment, treatment response, and transient short-term side effects. Detailed definitions can be found in Appendix B.

Using this classification, each assessed outcome was allocated to one of the defined outcome categories by two independent reviewers (N.S. and A.W.). The scores that the study reported were used to prioritize outcome categories to identify the highest and second-highest priority. The results were reported using descriptive data, describing the proportion of studies that prioritized each outcome category as highest or second highest in relation to the number of studies addressing that outcome.

If multiple outcomes in the study were allocated to the same outcome category (e.g., diarrhea and nausea were both transient short-term side effects), an average score of these outcomes was used to decide on the prioritization order of the outcome categories. In case of discrepancies, items were discussed until consensus was achieved; if needed, a third reviewer (M.H.) was consulted. When a study assessed preferred outcomes with multiple methods, resulting in different prioritizations, only the discrete choice elicitation was used to limit the heterogeneity. To determine the robustness of the results, subgroup analyses were conducted for curative and palliative settings and for older patients (study populations with a median age of 70 years or higher).

## 3. Results

### 3.1. Search and Study Selection

**Figure 1:**
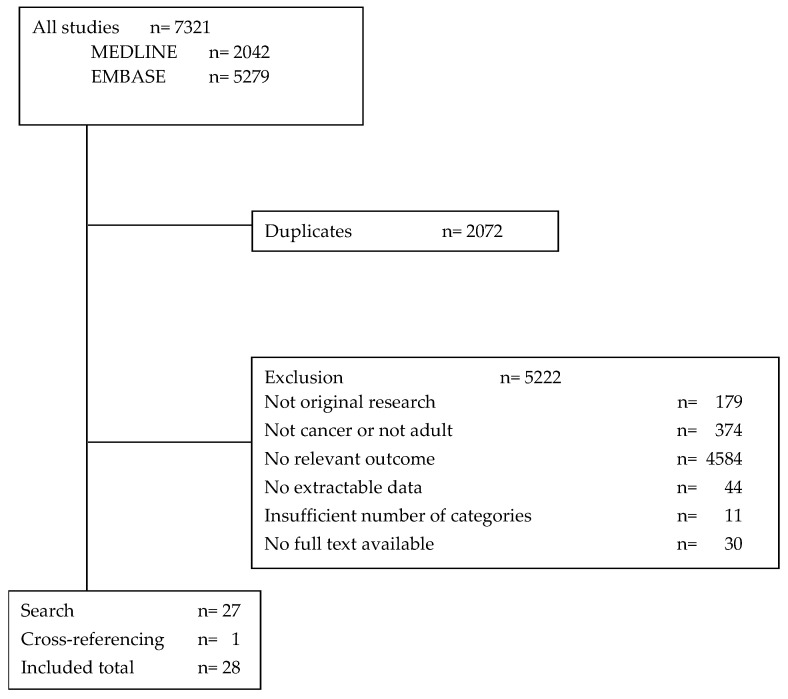
Study selection.

The search resulted in 7321 hits (2042 from Medline and 5279 from Embase). After removing 2072 duplicates and 5222 studies for other reasons (Figure 1), a total of 27 publications were included. Cross-referencing yielded one more publication, resulting in a total of 28 studies in this systematic review.

### 3.2. Study Characteristics

The characteristics of 28 selected studies are summarized in Table 1. Most were published in the past five years. The total study population consisted of 4374 patients; the median sample size was 133 patients (range 36–419). The me(di)an age of participants varied between 35 and 78 years and six studies had a population with a median age over 70 years. The most frequently studied cancer type was gastrointestinal cancer (*n* = 8), followed by six studies that assessed various cancer types (see Table 1). Eight studies examined curative treatment, 11 studied palliative treatment, and nine studied both. The studies that assessed both often had a mix of all stages of cancer together. Various methods of preference elicitation were used. The majority of the studies (*n* = 15) used discrete choice elicitation, followed by conjoint analysis (*n* = 5), the Outcome Prioritization Tool (*n* = 3), and various types of rating scales. Both probability trade-off and the analytic hierarchy process were used in one study (Table 2).

### 3.3. Quality Assessment

**Figure 2:**
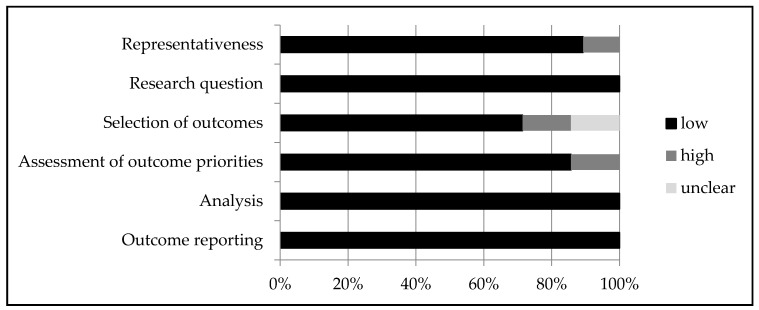
Quality assessment—risk of bias.

Figure 2 provides an overview of the quality assessment. Details of each study can be found in Appendix D. In general, the representativeness of patients was good, although a few studies asked patients to provide answers for a hypothetical situation—for example, what they would choose if they had a different type or stage of cancer [29,40]. Some studies did not clearly report how specific outcomes were selected [28,29], or did not describe selection procedures at all [41,47,48,50]. Additionally, sometimes it was unclear how quality of life or other attributes were defined or were described to patients [27,39]. The analysis and outcome reporting were heterogeneous, but overall well described.

### 3.4. Categories

The 28 publications reported 30 prioritizations: Two studies had a separate prioritization for patients with curative and palliative stages of disease. The median amount of outcome categories per study was four (range 3–6). The most frequently assessed outcome category was severe and persistent side effects (24 studies, 83%), followed by overall survival and transient short-term side effects (both, *n* = 19, 66%). Quality of life and progression- and disease-free survival were assessed in 14 (48%) and 15 studies (52%), respectively. Only one study included all six outcome categories (28), and there was no outcome category that was assessed in all studies.

### 3.5. Most Important Outcome Categories

**Figure 3:**
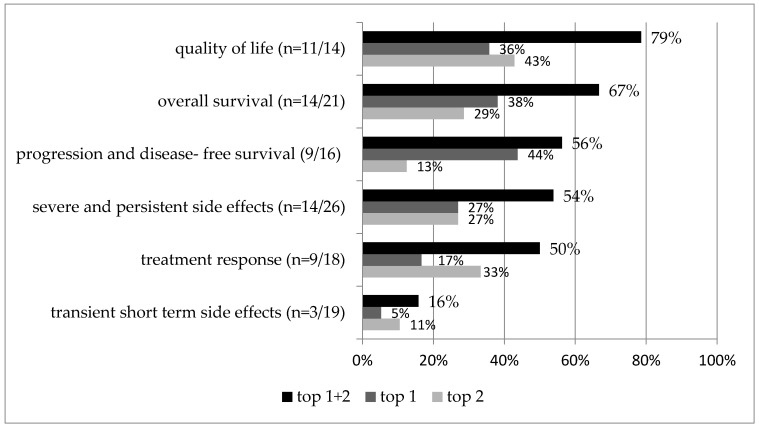
Outcome category prioritization of all studies.

**Figure 4:**
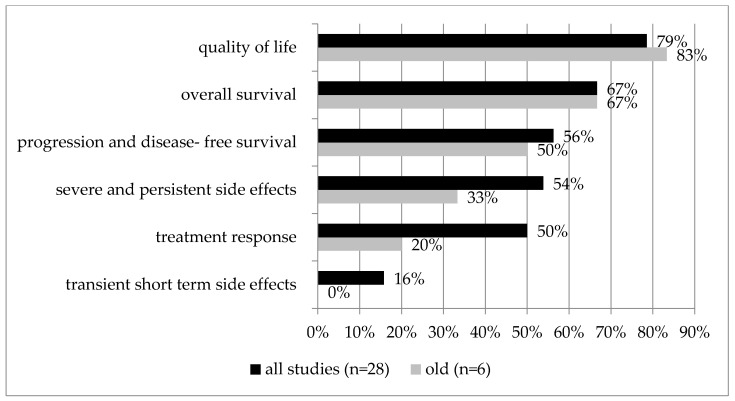
Older patient subgroup analysis. Outcome category prioritization of all studies (*n* = 28) compared to the subgroup of older patients (*n* = 6). A higher percentage means more frequently prioritized as the first or second priority. Percentages are relative to the number of studies that included that outcome category.

For each study, the highest and second-highest outcome categories are identified and shown in Figure 3 and Table 2 relative to the number of studies that assessed that outcome category. For example, quality of life was assessed in total in 14 studies and was in 11 studies the highest or second-highest priority (*n* = 11/14, 79%). Overall survival (67%), progression- and disease-free survival (56%), and severe and persistent side effects (54%) were also commonly prioritized. When focusing only on studies addressing a palliative setting, quality of life and overall survival were most important (both in 75%); in contrast, progression- and disease-free survival (67%) and treatment response (67%) were given the highest priority in a curative treatment setting (Table 2, Figure 4).

The higher the percentage, the more frequently that outcome category was prioritized. Top 1 and top 2 priorities are shown. Percentages are given relative to the number of studies that assessed that outcome category. In total, 30 rankings from 28 studies are included. *n* represents the number of rankings that assessed the category.

In a subgroup analysis of six studies focusing specifically on older patients [28,36,37,41,47,50] (median age of the study population of 70 years or higher or separate data of this subgroup), quality of life and overall survival were included in five of the six studies. They were also the highest or second-highest priority in most of them (*n* = 5/6, 83%, and *n* = 4/6, 67%, respectively), followed by progression- and disease-free survival (*n* = ½; 50%), severe and persistent side effects (*n* = 1/3; 33%), treatment response (*n* = 1/5; 20%), and transient short-term side effects (*n* = 0/2; 0%; see Table 2, Figure 4).

## 4. Discussion

In this systematic review, we examined which outcomes of treatment matter most to patients with cancer. While we were particularly interested in the priorities of older patients, the search was expanded because very few studies exist on patient preferences in older adults with cancer. In the 28 included studies, quality of life received high priority most frequently, followed by overall survival, progression- and disease-free survival, and severe and persistent side effects. In the palliative setting and in the subgroup analysis of older patients, quality of life and overall survival were most often prioritized, but in the curative setting, progression- and disease-free survival and treatment response were more important. This suggests that even though quality of life is most important overall on a group level, priorities might change depending on the intent of the treatment, contextual factors, and the age of the patient.

In this systematic review, in which the majority of the studies included younger patients, quality of life was often considered important. Although severe and persistent side effects were assessed in the majority of studies, quality of life was underappreciated and included only in half of the studies. These findings are in line with previous research [52,53,54,55,56,57,58]. In our subgroup analysis of older patients, quality of life was assessed in the majority (75%), and together with overall survival, most frequently prioritized (83%). The outcome category of quality of life included functional and other patient-centered outcomes, but often was described in an unspecific way. However, especially in older patients, specific components of quality of life like cognition and functional abilities are considered important and few patients are willing to trade cognition for survival [59,60]. Thus, due to the inclusion of all ages and the unclear descriptions of quality of life, our review might underestimate the importance of certain aspects of quality of life in older patients.

A recent systematic review on information needs in older patients with cancer showed that after patients received a cancer diagnosis, their focus was on short-term issues like understanding the situation, treatment options, and other practicalities, whereas information on functioning, quality of life, and dealing with late effects were given lower priority [6]. However, decision regret is more often linked to negative long-term outcomes [61], something that was discussed in half of the patients [62]. Since our study also shows that both quality of life (79%) and severe and persistent side effects (54%) were more frequently prioritized outcomes than transient short-term side effects (16%), patients should be aided in assessing and explicitly expressing which long-term outcomes matter most to them.

Although both patients and physicians consider efficacy and physical side effects important in treatment choice, patients also incorporate other factors in their decision-making, like the impact on their daily life, family responsibilities, and the ability to attend important life events [63]. In the translation of general treatment outcomes to their personal situation, patients might interpret the outcomes differently than the physician. In addition, they might not realize that one treatment might have multiple competing effects. If the patient’s interpretation is left unrevealed, this may lead to a treatment choice that may not provide the patient with the benefit that they desire, or may also lead to a negative effect that lessens the benefit. For example, a patient with metastatic cancer may state that extending survival is most important, with an unexpressed underlying desire to care for an ailing partner for as long as possible. If the physician then tailors the treatment to value extending life, intensive palliative chemotherapy may be started. Although this may increase survival, it may in fact also hamper the patient’s caregiving abilities due to the side effects of treatment.

These unique patient values that underlie the preference are not easily incorporated into disease- and treatment-specific decision aids. Moreover, physicians are not good at estimating their patients’ preferences [50]. Thus it can be helpful to have an additional preference assessment conversation as part of decision-making. This will help to clarify what a priority of a specific outcome means to the patient and why it is important to them, and will prevent treatment selection based on wrong interpretations from the patient or misunderstandings by the physician.

In our review, some studies [41,50] used a non-disease and non-treatment-specific generic communication tool developed for patients with multimorbidity by Terri Fried: the Outcome Prioritization Tool (OPT) [15]. During this conversation, the healthcare professional verifies whether he or she understands the trade-offs correctly and invites the patient to explain why the outcomes are important and how they were interpreted [14]. Although this tool has been used in oncology patients before [41,50,64], it might be worthwhile to adapt this tool specifically for cancer patients and the treatment decisions they have to make.

This study has some limitations. Firstly, the studies were heterogeneous; various types of cancer and various tumor stages were included. Furthermore, the studies assessed the priorities with their own defined benefits and risks. Depending on what was most appropriate given the characteristics of cancer-specific treatment regimens, they each asked the outcome categories differently and used different levels of the various attributes. Although the studies had sufficient common denominators to allow for the categorization and combination of results, this does make comparison between the palliative and curative treatment settings more difficult. For example, in a curative setting, overall survival might not be prioritized when described as a small increase in an already high survival rate. However, in the situation of a metastatic disease with a poor prognosis, overall survival might be prioritized, because living a few more months might be important for a patient who is awaiting their first grandchild.

Moreover, multiple methods of assessing these outcome preferences were used, all with their own benefits and risks of bias [13,65]. To be able to compare the various outcome categories relative to each other and to minimize the effect of chance on ending up as a high priority, only studies that assessed at least three categories were included. Studies comparing only two categories and studies where it was not possible to elucidate the relevance of the various outcome categories were excluded. This might have changed the results, but made comparisons possible and allowed an actual trade-off between the benefits and negative effects of treatments to be registered.

Finally, due to the heterogeneity of methods and reporting, we had to simplify the results of the studies to a ranking. This does not give details on whether the outcome priorities are close together or far apart within a study or on how much benefit gain or risk avoidance leads to a change in priority; thus, some information may have been lost. It did, however, allow us to identify the most important outcome categories on a group level. In clinical practice, these could be presented to individual patients who need to make and define their own trade-offs anyway. This pre-selection may prevent them from being overwhelmed by too many choices.

## 5. Conclusions

In conclusion, understanding how patients prioritize potential outcomes of oncologic treatment and the trade-offs they are willing to make is an important component of shared decision-making. Our systematic review shows that quality of life, overall survival, progression- and disease-free survival, and avoiding severe and persistent side effects of treatment are the outcomes that receive the highest priority in patients with cancer.

## Figures and Tables

**Table 1:** Included studies and their characteristics.

Author, Year	Country	*n*	% Male	Me(di)an Age (SD, Range or Percentage Older)	Type of Cancer	Treatment Setting
Pieterse [25], 2007	NL	66	68%	64 (±9)	Gastrointestinal	Curative
Mohamed [26] 2011	USA	138	49%	57 (±9)	Renal cell	Curative
Thrumurthy [27] 2011	UK	81	77%	67 (38% > 70)	Esophageal	Curative
* Jorgensen [28], 2013	Australia	68	100%	64 (51% > 65)	Colorectal	Curative
Molinari [29], 2014	Canada	75	61%	51 (±11)	HCC	Curative
Thill [16], 2016	Germany	41	100%	50 (29–76)	Breast	Curative
van der Valk [30] 2020	NL	94	43%	62 (±9)	Rectal	Curative
Werner [31], 2021	Germany	37	51%	59 (±9)	Anal/colorectal	Curative
Park [32], 2012	Korea	140	37%	57	Renal cell	Palliative
Havrilesky [33], 2014	USA	95	0%	60 (±10)	Ovarian	Palliative
DaCosta [34], 2014	USA	181	0%	52 (±9)	Breast	Palliative
Muhlbacher [35], 2015	Germany	211	65%	59 (±8)	NSCLC	Palliative
* Uemura [36], 2016	Japan	133	100%	75 (±7)	Prostate	Palliative
* Chau [37], 2016	Canada	36	100%	73	Prostate	Palliative
Gonzalez [38], 2017	USA	127	54%	46 (±16)	Colorectal	Palliative
Liu [39], 2019	USA	200	40%	34% >50	Melanoma	Palliative
Wong [40], 2020	Singapore	169	58%	62 (±11)	Colorectal	Palliative
* Stegmann [41], 2020	NL	53	72%	75 (±7)	Various types	Palliative
Weilandt [42], 2021	Germany	150	60%	59 (23–85)	Melanoma	Both
Johnson [43], 2006	USA	375	30%	61 (±12)	Various types	Both
Schmidt [44], 2017	Germany	310	62%	63 (±11)	Various types	Both
Sun [45], 2019	China	361	63%	58 (31–82)	NSCLC	Both
Bröckelmann [46], 2019	EU	289	64%	36 (19–75)	Lymphoma	Both
* Festen [47], 2019	NL	197	56%	78 (70–93)	Various types	Both
Valenti [48], 2020	Spain	100	51%	64 (29–85)	Various types	Both
Fifer [49], 2020	UK	419	44%	93% >50	MM	Both
* Festen [50], 2021	NL	87	52%	76 (IQR 72–80)	Various types	Both
Khan [51], 2020	USA	141	50%	35 (19–69)	Lymphoma	Both

NSCLC (non-small cell lung cancer), HCC (hepatocellular carcinoma), MM (multiple myeloma), NL (the Netherlands), USA (United States of America), UK (United Kingdom), EU (European Union). * Studies with median age above 70 or separate data for this subgroup.

**Table 2:** Ranking of outcome categories per study.

STUDY		Methods	Outcome Categories					
Author	Year	Elicitation Method	Quality of Life	Transient Short Term Side Effects	Severe and Persistent Side Effects	Treatment Response	Progression- and Disease-Free Survival	Overall Survival
Curative Setting Studies								
Pieterse [25]	2007	CA			5	4		3
Thrumurthy [27]	2011	DCE	5		3	4		2
Mohamed [26]	2011	DCE		3	4		5	
* Jorgensen [28]	2013	Rating scale	3	2	2		5	4
Molinari [29]	2014	Trade-off	2		5	4		3
Thill [16]	2016	AHP		2	3	5	4	
Bröckelmann [46]	2019	DCE		2	4		5	3
van der Valk [30]	2020	CA	4		5		3	3
Khan [51]	2020	DCE			5	3		4
Werner [31]	2021	Rating scale	4	2	1	3	3	5
Palliative Setting Studies								
Havrilesky [33]	2014	DCE		4	3	2	5	
DaCosta [34]	2014	CA	3	2	4			5
Park [32]	2012	DCE		5	4		3	
Muhlbacher [35]	2015	DCE		3	2	5	5	
* Uemura [36]	2016	DCE	4	1	5	2		3
* Chau [37]	2016	Rating scale	4		1	5	3	2
Gonzalez [38]	2017	DCE		3	5		4	
Liu [39]	2019	DCE			4	3	2	5
Bröckelmann [46]	2019	DCE		2	3		5	4
Wong [40]	2020	DCE		4	5		3	
Khan [50]	2020	DCE			4	3		5
* Stegmann [41]	2020	OPT	5			3		4
Weilandt [42]	2021	DCE		3	2	4	1	5
Both Settings Studies								
Johnson [43]	2006	CA	5	3	4			
Schmidt [44]	2017	DCE	4	3				5
Sun [45]	2019	DCE		2	3	4	5	
* Festen [47]	2019	OPT	5			3		4
Fifer [49]	2020	DCE		3	3	4		5
Valenti [48]	2020	CA	4	3	3			5
* Festen [50]	2021	OPT	5			3		4

Higher numbers represent higher priority (range 0–5). Where an outcome category is not assessed, no number appears. CA = conjoint analysis, DCE = discrete choice experiment, AHP = Analytic hierarchy process, OPT = outcome prioritization tool. * Studies with median age above 70 or separate data for this subgroup.

## Data Availability

The data presented in this study are available on request from the corresponding author.

## Appendix A. Search

The following search was performed on 22 December 2020, in both Embase and Medline (((“treatment*”[tiab] OR “health”[tiab]) AND “outcome*”[tiab]) OR “scenario*”[tiab] OR “vignet*”[tiab] OR (“research”[tiab] AND “agenda”[tiab])) AND ((“priorit*”[tiab] OR “preference*”[tiab] OR “trade”[tiab] OR “trade off*”[tiab]) AND (“patient”[tiab] OR “patients”[tiab]) AND (“cancer”[tiab] OR “oncolog*”[tiab] OR “malignan*”[tiab]). Limitations: humans, English or Dutch, 2006, and onward study selection.

## Appendix B

**Table A1:** Descriptions of the Outcome Categories.

Outcome Categories	Descriptions
Quality of life	Long-term maintenance of quality of life and functional status. Also includes other patient reported/centered measurements such as keeping one’s independence and social/role functioning. E.g., quality of life, maintaining independence of (instrumental) activities of daily living (iADL), worries, anxiety
Transient short-term side effects	Short-term and transient treatment-related toxicity/adverse events that terminate after cessation of treatment or that only require minimal medication. E.g., diarrhea, hair loss, nausea/vomiting, rash and skin change
Severe and persistent side effects	More severe adverse treatment events that require intensive treatment, hospitalization or discontinuation of treatment. Often patients will recover from these events, but it can take a long time (>6 months) and involves intensive treatment. Persistent side effects/sequelae inherent or caused by the treatment are also included here. E.g., severe bleeding, gastrointestinal perforation, heart attack, colostomy, neuropathy, fatigue, scars, permanent fecal incontinence, urinary incontinence, infertility
Treatment response	All treatment benefits, except for time-dependant measurements. Treatment benefits other than survival, like symptom reduction, response seen on scans, or risk reductions. E.g., symptom control, local control, complete response, partial response, recurrence risk
Progression- and disease-free survival	Progression-free survival or disease-free survival, but not overall survival. A time-dependent measurement that indicates how long the disease is under control or cured. Also a measurement of efficacy, but time-dependent. E.g., disease-free survival, progression-free survival, time to treatment failure
Overall survival	Overall survival and mortality independent of the cancer (treatment) at a certain time point during follow-up. E.g., overall survival, mortality at a certain time point, extending life

## Appendix C

**Table A2:** Quality assessment based on the Newcastle–Ottawa Scale.

selection	1. Representativeness of the cohort	+	Participants are patients who have cancer
		−	Participants are asked to answer as if they have cancer or as if they have a different stage of cancer
		?	The sampling strategy leads to non-representativeness
study design	2. Research question	+	Well-defined research question and hypothesis
		−	Vaguely described research question
		?	No research question present
	3. Selection of outcomes included	+	Clearly described why the different outcomes (= attributes) and levels are included in the study, e.g., based on previous research, pilot study, or qualitative research
		−	Described vaguely, but not clear why these items or levels are selected, e.g., in a discussion with researchers
		?	No description on why these outcomes are chosen
	4. Assessment of outcome priorities	+	Clear description of the definition of the outcomes and method of assessment and clear understanding of how the outcomes are described to the patient
		−	Unclear description of outcomes or unclear method of assessment, e.g., quality of life is mentioned without clear description
		?	No description of definitions and unclear method of assessment
outcome	5. Analysis	+	Clear description of method of analysis
		−	Unclear description of method of analysis
		?	No description
	6. Outcome reporting	+	Scores for all outcomes separately reported
		±	Outcomes reported, but hard to make a ranking or early stage and advanced stage of disease not separately reported
		−	Not all scores reported
		?	Unclear whether all outcomes are reported

## Appendix D

**Table A3:** Quality Assessment Per Study.

Author, Year	Representativeness	Research Question	Selection of Outcomes	Assessment of Outcome Priorities	Analysis	Outcome Reporting
Johnson, 2006	+	+	+	+	+	+
Pieterse, 2007	+	+	+	+	+	+
Thrumurthy, 2011	+	+	+	−	+	+
Mohamed, 2011	+	+	+	+	+	+
Park, 2012	−	+	+	+	+	±
Jorgensen, 2013	+	+	−	+	+	+
Havrilesky, 2014	+	+	+	+	+	+
DaCosta, 2014	+	+	+	+	+	+
Molinari, 2014	−	+	−	+	+	+
Muhlbacher, 2015	+	+	+	+	+	+
Uemura, 2016	+	+	+	+	+	+
Thill,2016	+	+	+	+	+	±
Chau, 2016	+	+	+	+	+	+
Gonzalez, 2017	+	+	+	+	+	+
Schmidt, 2017	+	+	+	+	+	+
Bröckelmann, 2019	+	+	+	+	+	+
Sun, 2019	+	+	+	+	+	+
Liu, 2019	+	+	+	−	+	+
Festen, 2019	+	+	?	+	+	+
van der Valk, 2020	+	+	+	+	+	+
Valenti, 2020	+	+	?	+	+	±
Wong, 2020	−	+	+	+	+	+
Stegmann, 2020	+	+	?	+	+	±
Fifer, 2020	+	+	+	−	+	+
Khan, 2020	+	+	−	+	+	+
Festen, 2021	+	+	?	+	+	+
Werner, 2021	+	+	+	+	+	+
Weilandt, 2021	+	+	−	−	+	+
